# Parental experience of neonatal pain research while participating in the Parental touch trial (Petal)

**DOI:** 10.1097/j.pain.0000000000003177

**Published:** 2024-01-25

**Authors:** Marianne van der Vaart, Annalisa G.V. Hauck, Roshni Mansfield, Eleri Adams, Aomesh Bhatt, Maria M. Cobo, Daniel Crankshaw, Amraj Dhami, Caroline Hartley, Vaneesha Monk, Ria Evans Fry, Fiona Moultrie, Shellie Robinson, Jean Yong, Ravi Poorun, Luke Baxter, Rebeccah Slater

**Affiliations:** a Department of Paediatrics, University of Oxford, Oxford, United Kingdom; b Newborn Care Unit, John Radcliffe Hospital, Oxford University Hospitals NHS Foundation Trust, Oxford, United Kingdom; c Colegio de Ciencias Biologicas y Ambientales, Universidad San Francisco de Quito USFQ, Quito, Ecuador; d University of Exeter Medical School, Exeter, United Kingdom; e Children's Services, Royal Devon University Healthcare NHS Foundation Trust, Exeter, United Kingdom

**Keywords:** Pain management, Parent–child relations, Motivation, Surveys and questionnaires, Emotions

## Abstract

Parental involvement in neonatal comfort care is a core component of family-centred care. Yet, parents experience a range of positive and negative feelings when providing pain-relieving interventions for their infants. Parents of infants who participated in the Parental touch trial (*Petal*), a multicentre randomised controlled trial investigating the impact of gentle parental touch on neonatal pain, were asked to complete an anonymous survey. This survey aimed to (1) explore parent-reported motivations in deciding to participate in the Petal trial; (2) understand parent-reported experiences related to trial participation; (3) understand parents' willingness to participate in future studies; and (4) evaluate parent-reported feelings while they were delivering a gentle touch intervention either before or after a clinically necessary blood test. One hundred six parents (1 parent per infant) took part in the survey. Primary motivators for participation were altruistic. Parents most frequently reported that they wanted their child to take part in the research because it has a potential benefit to babies in the future and because they wanted to improve scientific understanding. Parents reported that providing gentle touch to their children during painful procedures was associated with positive emotions, such as feeling “useful” (64%) and “reassured” (53%). Furthermore, nearly all parents (98%) were pleased to have participated in the Petal trial and would consider, or maybe consider, participating in further research studies. These results underscore the importance of structuring trials around parental involvement and providing opportunities for parents to be involved in providing comfort to their infants during necessary painful clinical procedures.

## Abstract 2

Parents report positive experiences when being involved in their baby's comfort care as part of a clinical trial.

## 1. Introduction

Many babies require painful clinical procedures soon after birth, either as part of their care in a neonatal unit^7,9^ or for routine blood tests^26^ and vaccinations.^13^ This not only affects the individual infants but also their families because concerns about infant pain are a source of parental stress.^16,18,19,25^

Parental involvement in neonatal pain relief is one of the core elements of family-centred care^4^ and can help parents to establish their caregiver role.^17,35^ Parents can use a wide-range of comforting techniques to support their infants during painful procedures, such as breastfeeding,^34^ kangaroo care,^24^ and facilitated tucking.^20^ Although previous studies have consistently highlighted the desire of parents to be involved in their child's comfort care,^29,39^ parents sometimes report experiencing feelings of anxiety or discomfort when attempting to deliver comforting interventions.^1,35^ A recent review concluded that there is a lack of research regarding parental motivations and experiences in delivering care, which should be addressed to improve parental involvement in the Neonatal Intensive Care Unit (NICU).^39^

It is vitally important that parents are given the opportunity to provide comfort to their infants, and parents should be considered as key partners in pain management strategies. The Parental touch trial (*Petal*)^8^ investigated the impact of gentle parental touch on procedural pain in newborn infants and on parental anxiety. No difference in neonatal pain outcome measures or parental anxiety was observed in relation to whether the parental touch was delivered shortly before (intervention group) or after (control group) a clinically necessary blood test.^8,22^ The trial provided a further opportunity to understand parental motivations to allow infants to participate in neonatal research and to explore their experiences of delivering comfort care during painful clinical procedures.

Following trial participation, we asked parents to complete an anonymous survey to explore their experience of participating in the trial. Here, we report the results of the survey and describe (1) the parent-reported motivations in deciding to enrol in the trial; (2) the parent-reported experience of participating in the trial; (3) parental willingness to participate in future studies; and (4) parent-reported feelings while they delivered the gentle touch intervention either before or after the clinically necessary painful procedure.

## 2. Methods

The aim of this work was to explore parental anxiety and distress, and parents' experience while taking part in a multicentre randomised controlled trial investigating the effect of parental touch on neonatal pain. Exploration of these factors was specified in the preregistered trial protocol as an exploratory objective.^8^

### 2.1. Ethics and recruitment

This survey was approved as one of the components of the Petal trial by the London—South East Research Ethics Committee (ref: 21/LO/0523). The trial is registered with ISRCTN (identifier ISRCTN14135962) and ClinicalTrials.gov (identifier NCT04901611). The Petal trial was conducted at the John Radcliffe Hospital (Oxford, United Kingdom) and Royal Devon and Exeter Hospital (Exeter, United Kingdom). Parents of eligible infants were given verbal and written information about the study, and written informed consent was obtained. The local hospital charity Supporting the Sick Newborn And their Parents (SSNAP) reviewed parent-facing materials. Parents did not receive financial remuneration for taking part in the study.

### 2.2. Design

The Petal trial is a multicentre, randomised, controlled trial investigating the effect of parental touch on neonatal procedural pain relief and parental anxiety during a clinically required heel lance. A detailed overview of the study design, including sample size planning, and study procedures has been published.^8^ In brief, infants were randomised (allocation ratio 1:1) to receive parental touch either before the clinically required heel lance (intervention group) or shortly afterwards (control group). Before, during, and after the clinical procedure, electroencephalography (EEG) and vital signs (electrocardiography, plethysmography) were continuously recorded. Parental anxiety before and after the clinical procedure was assessed using the State component of the State Trait Anxiety Inventory (STAI-S).^36^

The touch intervention involved one parent stroking their baby's lower leg ipsilateral to the site of the heel lance. Guided by a computer animation displayed on a screen, parents stroked their baby's leg for 10 seconds at a speed of approximately 3 cm/second. The animation included a 3-second countdown timer and a progress bar extending 3 times over a 10-cm distance at a speed of 3 cm/second for a total duration of 10 seconds. Before commencing the stroking intervention, parents demonstrated to the researchers their ability following the animation and performing the stroking according to the trial's protocol. In the intervention group, parents stroked their baby's leg just before the heel lance, whereas in the control group, they stroked their baby's leg after the heel lance.

Parents could talk or sing to their baby during the intervention if they wished to; however, we did not systematically record whether they provided any other sensory stimuli besides the parental touch intervention.

The parent who performed the intervention was asked to complete an anonymous survey at the end of the test occasion. These data are decoupled from the data of the rest of the study, including demographic data. Parents were presented with the online survey on an electronic device by a member of the research team. Responses were collected on Jisc Survey (Jisc, Bristol, United Kingdom, https://www.onlinesurveys.ac.uk). Survey questions were developed and piloted in an independent ongoing study (Investigating Pain in the Developing Brain; ethics ref: 12/SC/0447) to assess appropriateness and then tailored to this study. The survey was conducted in English. The first part was completed by the researcher to document the research site (Exeter, Oxford) and trial arm (parental touch before the heel lance or parental touch after the heel lance). The second part was completed by the parent and included 4 compulsory questions:

(1) “How important were the following factors in helping you decide to take part in the Petal trial?” (5 options, which could be graded as very important/important/neutral/not very important/not considered);
  (a) “Contributing to science”
  (b) “The potential benefit to your baby”
  (c) “The potential benefit to future babies”
  (d) “Knowing that your baby would be monitored closely by the trial team during the blood test”
  (e) “Concern that your baby might be in pain from the blood test”
(2) “Being actively involved in my child's care at the time of the blood test made me feel…” (a list of descriptors where multiple options could be selected out of a list of 10 adjectives): “anxious,” “stressed,” “relaxed,” “empowered,” “helpless,” “calm,” “frustrated,” “useful,” “upset,” “reassured.”
(3) “Are you pleased that your baby took part in the Petal trial?” (Yes/No);
(4) “Would you consider taking part in future research studies?” (Yes/Maybe/No).

### 2.3. Inclusion and exclusion criteria

The trial's inclusion and exclusion criteria are listed in Table 1. Between September 2021 and February 2023, 159 parents were approached about participation in the Petal trial. A total of 114 agreed to participate, and 112 babies participated in the trial (2 babies became ineligible before randomisation).

**Table 1:** Petal trial eligibility criteria.^8^

Inclusion criteria	Exclusion criteria
Participants born at the John Radcliffe Hospital, Oxford, or the Royal Devon and Exeter Hospital, Devon	Hypoxic ischaemic encephalopathy
Neonates born at or after 35 + 0 wk gestation	Intraventricular haemorrhage > grade II
Neonates with a postnatal age of ≤7 d	Received any analgesics or sedatives in the past 24 h
Neonates who require a heel lance as part of clinical care	Congenital malformation or genetic condition known to affect neurological development
Neonates for whom parents/guardians have given written informed consent for participation	Born to mothers who have a history of substance abuse

The inclusion and exclusion criteria refer to the babies.

### 2.4. Analysis

Descriptive statistics (percentage of respondents for each multiple-choice answer) are reported for each survey question. The network plot was produced in R^31^ using publicly available code.^33^ For survey question 2, differences in the frequency of each parent-reported feeling between trial arms were explored using Fisher exact test of contingency at an alpha level of 0.05 implemented in MATLAB (Mathworks, version 2022b). As the analysis was exploratory, no correction for multiple comparisons was made. Correlations between parent-reported feelings were explored using Pearson correlation coefficients and hierarchical clustering using Ward method with Euclidian distances, implemented in MATLAB (Mathworks, version 2022b).

To assess the change in parental anxiety after the clinical procedure (regardless of trial arm), STAI-S change scores were computed for each parent. A general linear model (GLM) was used to calculate the mean change in STAI-S scores, adjusted for STAI-S score before the procedure. Differences in anxiety change scores between mothers and fathers, adjusted for STAI-S scores before the procedure, were explored using a GLM. As the residuals of the models were not normally distributed, *P* values for both GLMs were derived nonparametrically using the tool Permutation Analysis of Linear Models (PALM^42^) with 10,000 permutations. The difference in parental anxiety between trial arms is reported elsewhere.^22^

## 3. Results

### 3.1. Demographic characteristics of participants in the petal trial

A total of 112 parent–infant dyads participated in the Petal trial, of which 106 parents completed the anonymous survey after their participation in the trial (2 parents did not complete the survey, and 4 infants were withdrawn). STAI-S scores before and after the clinical procedure were available for 106 parents. Table 2 provides the demographic characteristics of the infants enrolled in the trial.

**Table 2:** Demographic characteristics for all babies enrolled in the petal trial (n = 112).

Baseline characteristic	Stroking preprocedure (n = 56)	Stroking postprocedure (n = 56)
Parent stroking*		
Biological father	20 (36%)	19 (34%)
Biological mother	36 (64%)	37 (66%)
Gestational age at birth (wk)	38.52 (2.07)	38.28 (1.97)
Postmenstrual age at time of study (wk)	38.95 (2.02)	38.70 (1.95)
Postnatal age at time of study (d)	2.96 (1.82)	2.98 (1.90)
Birthweight (g)	3298 (653)	3201 (626)
Sex		
Female	22 (39%)	22 (39%)
Male	34 (61%)	34 (61%)
Mode of delivery		
Normal vaginal	23 (41%)	20 (36%)
Breech vaginal	1 (2%)	0
Elective C-section	14 (25%)	9 (16%)
Emergency C-section	12 (21%)	15 (27%)
Ventouse/forceps	6 (11%)	12 (21%)
Apgar score at 1 minute	8.31 (1.89)	8.48 (2.15)
Apgar score at 5 minutes	9.65 (0.69)	9.44 (1.11)
Primary reason for blood test		
Glucose monitoring	3 (5%)	3 (5%)
Jaundice	26 (46%)	28 (50%)
Newborn screening	4 (7%)	4 (7%)
Suspected sepsis	17 (30%)	16 (29%)
Other	6 (11%)	5 (9%)
Site		
Exeter	15 (27%)	14 (25%)
Oxford	41 (73%)	42 (75%)
Ethnicity		
White	50 (89%)	41 (73%)
Asian, Asian British, Asian Welsh;Black, Black British, Black Welsh, Caribbean or African; Mixed or Multiple; or Other ethnic group	6 (11%)	14 (25%)
Estimated cumulative prior pain exposure	4.68 (4.59)	4.69 (4.34)

Data are mean (SD) or count (%).

Estimated cumulative prior pain exposure refers to the cumulative number of skin-breaking blood tests, oral suctions, and endotracheal suctions. Ethnicity groups correspond to the 5 high-level ethnic groups used in the census for England and Wales^28^; ethnicity group of 1 participant is missing.

* In the 4 cases where the infant was withdrawn before the intervention, the parent who had planned to perform the intervention is indicated.

### 3.2. Parental motivation for taking part in the petal trial and in future research trials

**Figure 1.**
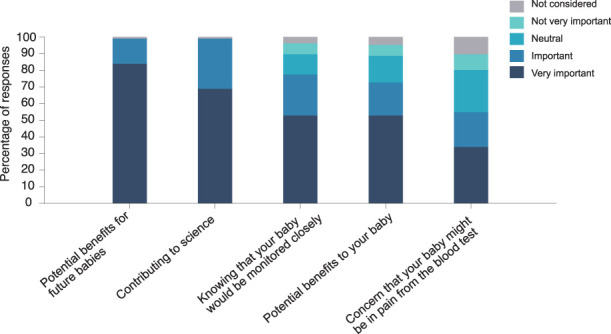
The importance of 5 factors that influenced parents' decision to take part in the Petal trial. Parents rated each of the 5 factors as “very important,” “important,” “neutral,” “not very important,” or “not considered.” The plot shows their reasons for taking part in the trial, sorted by the proportion of parents who found the factor important or very important. Potential benefits to future babies and contributing to science were rated as most important in the decision to take part, but individual factors (additional monitoring, potential benefits, and concerns about pain) were also considered “important” or “very important” by most parents.

Parents were asked to rate whether 5 factors were relevant when considering their decision to allow their infant to take part in Petal. The survey identified that the 2 primary motivators were (1) the potential benefit to babies in the future and (2) contributing to science, which were both considered “very important” or “important” by 99% of parents. Figure 1 summarises the relative importance of each of the 5 factors related to the parents' decision to take part in the study.

Factors directly relating to care needs for their baby also influenced parents' decision to take part, albeit to a lesser extent. Overall, 73% of parents considered the potential benefit to their baby to be “very important” or “important,” and 77% of parents considered the additional clinical observations that were provided as part of the trial (which included continuously monitoring the infants' heart rate, breathing rate, and oxygen saturation during the heel lance procedure) as “very important” or “important.” Concerns that their baby might be in pain was a “very important” or “important” motivator for just more than half of parents (55%).

Overall, 98% of parents were pleased that they took part in the Petal trial, and 98% said that that they would consider (75%) or maybe consider (24%) taking part in future research trials.

### 3.3. Parental experiences of being involved in their child's care at the time of the blood test

As part of the Petal trial, parents were asked to gently stroke their baby either before or after a heel lance that was being undertaken to obtain a clinically required blood sample. Parents were given the opportunity to select up to 10 words that best described their experience in being involved in their child's care at the time of the blood test. Parents commonly chose positive descriptors, and most commonly reported feeling (1) “useful” (64%), (2) “reassured” (53%), and (3) “calm” (44%).

**Figure 2.**
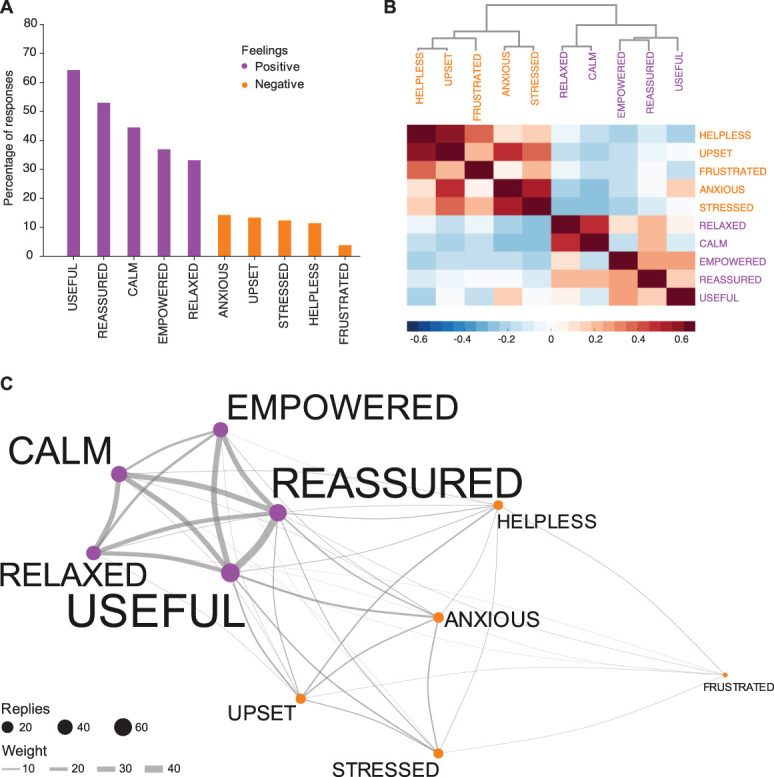
Parent-reported feelings about being actively involved in their child's care at the time of the blood test. Parents were asked to select up to 10 descriptive words to describe their experience. (A) Percentage of parents who reported each feeling, ranked from the most commonly selected feeling to the least commonly selected feeling. Positive feelings (purple) were selected more often than negative feelings (orange). (B) Dendrogram and correlation matrix showing the correlations between parent-reported feelings during the clinical procedure. Negative feelings (orange, left) and positive feelings (purple, right) are clustered. Colour bar indicates Pearson correlation coefficient. Clustering was performed using Ward method based on Euclidean distances. (C) Network graph depicting the descriptive words that were chosen together. The size of the nodes (circles) represents how many times parents chose each adjective as an answer. The weights of the lines reflect how often each pair of adjectives was chosen in combination.

Negative descriptors such as feeling (1) “frustrated” (4%), (2) “helpless” (11%), and (3) “stressed” (12%) were chosen much less often (Fig. 2A). In general, the positive and negative descriptors formed separate data clusters (Figs. 2B and C); however, many parents who reported one or more negative emotions (eg, feeling “anxious”) also reported at least 1 positive emotion (Fig. 2C). This highlights that clusters of positive and negative feelings are not completely exclusive, and parents who may have felt stressed or anxious can also concomitantly report feeling useful and reassured.

Parental anxiety, as assessed by STAI-S score, significantly decreased following the clinical procedure during which parents were actively involved (GLM, n = 106, mean STAI-S change score = −3.07, t-statistic = −4.77, *P*-value = 0.0001). Change in anxiety did not differ between mothers and fathers (GLM, mothers n = 70, fathers n = 36, adjusted mean difference in STAI-S change score (mothers − fathers) = 0.97, t-statistic = 0.70, *P* value = 0.44).

**Figure 3.**
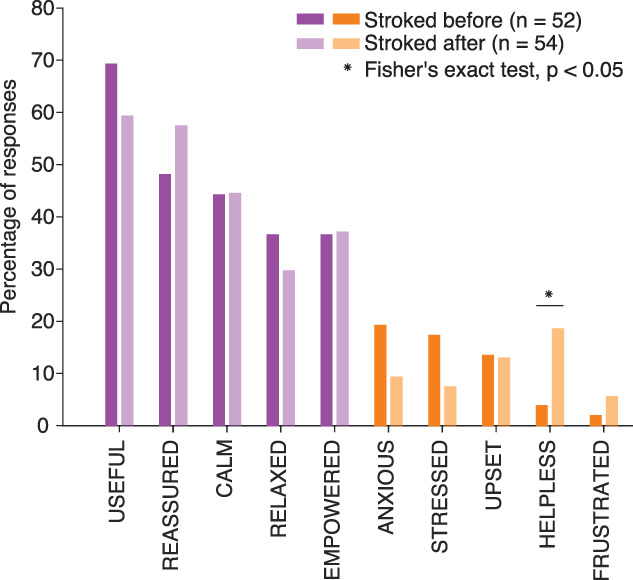
Percentage of parents who reported each feeling, split by trial arm. There were no significant differences between the intervention and the control groups, except for the feeling of helplessness, which was more prevalent in the postprocedural stroking control group. As this was an exploratory analysis, no correction for multiple comparisons was applied.

Overall, parents reported similar feelings regardless of whether they were asked to stroke their baby before or after the heel lance (Fig. 3). The descriptor “helpless” was more frequently selected by parents in the control group who provided reassuring tactile stimulation after the heel lance (n = 54) compared with those in the intervention group who provided preemptive gentle touch before the clinical procedure (n = 52, Fisher exact test, *P* = 0.03, Fig. 3, not corrected for multiple comparisons).

## 4. Discussion

Parental involvement in newborn care is a key component of neonatal pain management. The Petal trial was designed to explore whether stroking could reduce neonatal pain, and it provided an excellent opportunity to better understand the parental perspectives related to their involvement in a clinical trial and in providing pain-relieving interventions for their child during a clinically required blood test. As part of the trial, parents were asked to complete an anonymous survey related to their experiences of taking part in research investigating touch as a neonatal pain-relieving intervention, where parents had an active role in their child's care.

Nearly all surveyed parents (98%) were pleased that they took part and stated that they would potentially consider taking part in future trials. This is highly encouraging because it demonstrates the willingness of parents to engage in research to improve child health and the acceptability of the additional clinical monitoring that was an inherent part of the trial design. The survey was anonymous to encourage parents to respond candidly, and the response rate was nearly 100% (106 of 108 parents who completed the trial procedures completed the survey).

Of the 159 parents who were approached about participation in the Petal trial, 114 (72%) agreed to take part, which is consistent with other studies.^12,41^ We found that parents were primarily motivated to participate in the Petal trial for altruistic reasons, such as the advancement of scientific knowledge and the potential benefit to babies in the future, which supports previous research in this area.^5,11,21,27^ Most parents were also motivated because their baby would be closely monitored during the study period and because they felt that there were potential direct benefits for their baby—these factors have also been reported as motivators in previous neonatal studies.^10,41^ Concerns about their own child's pain was reported as an important motivator for approximately half of the surveyed parents. Our survey was not administered to parents who declined participation (45 parents, 28%) and therefore cannot identify potential barriers to consent. Examples, such as concerns about study logistics and procedures and feeling too overwhelmed to make decisions have previously been reported.^41^ It is also important to note that these findings cannot necessarily be generalised to all neonatal studies, such as clinical trials of pharmacological analgesics, where motivations and concerns regarding trial participation could substantially differ.

This study recruited near-term and full-term newborn infants in the first days of life. Understanding parental views about neonatal pain is important because how we manage and treat pain in early life can shape future pain experiences.^37,38,40^ Engaging with parents about optimal approaches to treat pain in early childhood provides an opportunity to educate families about how best to manage pain during subsequent procedures, such as routine blood tests or immunisations. The parental views expressed in this study cannot be directly extrapolated to families of preterm babies or babies who require prolonged hospitalisation. Given that infants receiving intensive neonatal care are exposed to an average of 16 heel lances in the first 2 weeks of life,^9^ as well as other painful procedures,^7,32^ it is likely that these parents' perspectives will differ from those surveyed in this study.

Parental presence and participation in neonatal care during clinical procedures can be associated with parental anxiety and stress.^15,30^ In the Petal trial, we observed that only a minority of parents reported 1 or more negative feelings while being involved in their child's care during the blood test. Our survey was not set up to explore the underlying reasons for this or the relative contributions of the various factors that might influence these emotions, such as the nature of the clinical procedure studied here, parental concern about their child's pain, or the reason the blood test was needed. Future targeted qualitative research could carefully explore how such factors might lead to parental anxiety when taking part in their baby's comfort care during procedures. This knowledge could help to ensure that appropriate targeted support can be provided for parents depending on their needs. Reassuringly, there was an overall decrease in parental anxiety following the blood test. This suggests that despite observing the painful clinical procedure, parents were not more anxious following their involvement in their child's comfort care than before. Because the trial did not include an arm where parents were not present for the clinical procedure, this decrease in anxiety cannot be directly attributed to the study intervention. However, this observation indicates that it is important not to assume that presence during clinical procedures will heighten parental anxiety. Furthermore, some parents who felt helpless, frustrated, anxious, or stressed also reported feeling useful, suggesting the experiences associated with supporting a child during painful procedures can be mixed. This is supported by previously reported findings, in which some parents felt uncomfortable when delivering a parent-led facilitated tucking intervention but at the same time wanted to be involved in their child's care.^1^ The timing of parental involvement may also influence negative perceptions. In our exploratory analysis, we observed that feelings of “helplessness” were less prevalent in the group of parents who performed the parental stroking intervention before the clinical procedure, as compared with the parents who performed the parental stroking afterwards. This is an important preliminary observation that warrants further investigation because it implies that parents may feel a positive sense of involvement when they provide preemptive pain relief for their child before painful procedures are performed.

A strength of the Petal trial is that it involved both fathers and mothers. In this study, 65% of the infants received the parental intervention from their mother and 35% of infants received the parental intervention from their father. Previous studies have indicated that engagement with fathers in a NICU setting is still suboptimal and should be improved^6,14^ because fathers have expressed feelings of helplessness and a desire to be present for their child.^23^ As such, it is important that both fathers and mothers are given the opportunity to be involved in neonatal research, and it is encouraging that both mothers and fathers were willing to be involved in this trial, and on balance, they found the experience positive. In addition, the mean reduction in anxiety following the blood test did not differ between mothers and fathers, suggesting that the experience was comparable for both. The anonymous survey did not identify whether the father or the mother completed the questions, and therefore, other differences in their experiences were not further explored here.

In conclusion, our results show that parents had an overwhelmingly positive experience while participating in the Petal trial, which investigated parental touch as a pain-relieving intervention for neonates. The findings highlight the importance of including parents in neonatal pain research and the value in understanding their experiences related to the provision of parent-delivered pain-relieving interventions. This work supports international guidelines, which highlight the importance of informing and involving families in the management of neonatal pain and stress.^3^ Future studies should aim to further understand the barriers to parental involvement in comfort care,^30^ so that the partnership between families and healthcare providers can be strengthened.^2^ It is highly encouraging that parents overwhelmingly described the experience of taking part in the Petal trial as positive. The work highlights the importance parents give to adequate pain provision for their children and the desire for them to contribute to this important area of research.

## Conflict of interest statement

This work is supported by Bliss (a UK charity supporting neonates and parents) via a research grant and by a Wellcome Senior Fellowship awarded to Rebeccah Slater (grant number 207457/Z/17/Z). Bliss is developing a campaign to advise parents and health care professional how parents can support their babies during painful procedures.
